# Analysis of the Correlation Between the Expression of T-Helper Type 17 Cell-Related Cytokines and Valve Damage in Rheumatic Heart Disease

**DOI:** 10.7759/cureus.72759

**Published:** 2024-10-31

**Authors:** Fraz Ahmad, Abdur Raheem Arshed, Muhammad Habib Mumtaz, Fatima Amjad, Maryyam Tariq, Adeeba Fatima, Bilal Qammar, Maryyam Islam, Maryam Ahmad, Hassam Ali

**Affiliations:** 1 Cardiology Department, Shalamar Hospital, Lahore, PAK; 2 Internal Medicine Department, Mayo Hospital, Lahore, PAK; 3 Internal Medicine Department, Werribee Mercy Hospital, Melbourne, AUS; 4 Internal Medicine Department, Shalamar Hospital, Lahore, PAK; 5 Internal Medicine Department, District Head Quarter Teaching Hospital, Mirpur, PAK; 6 Medicine Department, Shalamar Hospital, Lahore, PAK; 7 Research and Innovation Department, Shalamar Medical and Dental College, Lahore, PAK; 8 Medicine and Surgery Department, Shalamar Medical and Dental College, Lahore, PAK

**Keywords:** inflammatory response, mitral valve replacement, rheumatic heart disease, th17 cells, valve damage

## Abstract

Introduction: Rheumatic heart disease (RHD) results from chronic inflammation and fibrosis of heart valves following untreated rheumatic fever, yet its immunopathology, particularly involving T helper 17 (Th17) cells and their cytokines, is not fully understood. Th17 cells are prominent drivers of inflammation and have been linked to various autoimmune diseases, suggesting their potential role in RHD-related valve damage. This study examines Th17-associated cytokines-interleukin (IL)-17, IL-6, IL-23, and IL-21-in RHD. IL-17 is known to amplify inflammation by inducing pro-inflammatory cytokines and recruiting neutrophils, likely exacerbating valve damage, while IL-6 plays a role in Th17 differentiation and may promote chronic inflammation linked to fibrosis. IL-23 sustains Th17 cells, thereby perpetuating the inflammatory cycle in RHD, and IL-21 influences Th17 cells and B cell responses, potentially linking adaptive immunity to valve pathology. Clarifying the roles of these cytokines could offer insights into novel therapeutic targets for mitigating inflammation and preventing disease progression in RHD.

Methods: This study included 20 patients with RHD undergoing mitral valve replacement (Group O) and 20 patients with degenerative mitral valve prolapse (Group C) as controls. We utilized immunohistochemical staining and hematoxylin and eosin staining to assess the expression of Th17 cell-related cytokines in heart valves. To explore the relationship between cytokine expression and valve damage, Spearman correlation analysis was conducted. For inter-group comparisons, we employed the Mann-Whitney U test and Wilcoxon rank-sum test for non-parametric data, facilitating the evaluation of significant differences in cytokine levels and other relevant variables.

Results: In Group O, the percentage of IL-17-positive cells in mitral valve tissue was 51.6 ± 18.4%, with an immunohistochemistry score of 3.7 ± 2.2. IL-21-positive cells constituted 54.2 ± 16.5% with a score of 5.2 ± 1.3, IL-6-positive cells were at 29.4 ± 17.3% (score of 3.9 ± 1.5), and IL-23-positive cells accounted for 33.7 ± 17.9% (score of 4.3 ± 1.6). All these cytokine levels were significantly higher in Group O compared to Group C (P < 0.05), and the expression levels of IL-17, IL-21, IL-6, and IL-23 in valve tissue positively correlated with heart valve damage (P < 0.05). The main findings indicated that patients with RHD had significantly elevated levels of pro-inflammatory cytokines, including TNF-α, IL-6, and IL-10, compared to healthy controls. These elevated cytokine levels were associated with the severity of valve damage in RHD patients, suggesting a critical role of the inflammatory response in the progression of valve injury; specifically, higher concentrations of TNF-α correlated with more severe mitral regurgitation, while increased levels of IL-6 were linked to a higher grade of mitral stenosis.

Conclusion: The study reveals a significant association between Th17 cell-related cytokine expressions and valve damage in RHD patients, suggesting that targeting these cytokines may offer a promising strategy for treatment and prevention while highlighting their potential as biomarkers for assessing valve damage and the importance of managing inflammation to mitigate further cardiac complications.

## Introduction

Rheumatic heart disease (RHD) is a severe cardiac condition whose pathogenesis is associated with untreated streptococcal infections, typically pharyngitis or tonsillitis during childhood [1]. Although the incidence of RHD has significantly declined in developed countries due to widespread antibiotic use and improved healthcare conditions, it remains a substantial public health concern in some developing nations. Globally, RHD affects millions of individuals, with an estimated prevalence of 15.6 million cases and approximately 300,000 deaths annually, particularly in regions such as sub-Saharan Africa and South Asia, where it remains endemic [2]. Patients with RHD often present with symptoms such as cardiac murmurs, dyspnea, fatigue, and edema [3]. In severe cases, RHD can lead to heart failure, arrhythmias, or even sudden death [4]. RHD primarily affects cardiac valves, resulting in persistent inflammation and structural changes in the mitral and aortic valves [5]. This valve damage can lead to valve stenosis or regurgitation, ultimately impairing cardiac function. Pathologically, valvular damage in RHD exhibits several characteristics: continuous valve inflammation leading to valve thickening, stiffening, and deformation; the presence of fibrous nodules (Aschoff nodules) on the valves, which is a hallmark of RHD; valve calcification, increasing the risk of valve stenosis; and valve regurgitation, causing backflow in the heart valves [6,7]. Valvular damage in RHD significantly impacts patients' quality of life and may result in heart failure, arrhythmias, and other severe cardiovascular complications [8].

Th17 cells represent a novel subset within the CD4+ T cell population [9]. Overactive Th17 cells are associated with various conditions, including rheumatoid arthritis and inflammatory bowel disease [10]. Th17 cell-related cytokines have garnered significant attention in the field of immunology, as they play crucial roles in multiple physiological and pathological processes, such as inflammation, immune regulation, and autoimmune diseases [11]. Among these, cytokines such as interleukin (IL)-17, IL-6, IL-21, and IL-23 exert key regulatory functions in the differentiation and function of Th17 cells. IL-17 is the prototypical secretory cytokine of Th17 cells and plays a pivotal role in initiating and sustaining inflammatory responses by promoting inflammatory cell infiltration and inducing the release of other inflammatory cytokines [12]. IL-6 promotes Th17 cell differentiation and functions in activating other immune cells and inflammatory responses [13]. Research indicates that IL-21, secreted by natural killer cells, can induce Th17 cell differentiation in the absence of IL-6, highlighting its critical importance for the maintenance of Th17 cells [14]. While IL-23 is not required during the initial stages of Th17 cell differentiation, it becomes essential for regulating and maintaining Th17 cell effector functions, as it induces IL-22 secretion by Th17 cells, amplifying and stabilizing the Th17 cell phenotype [15].

In the context of heart valve damage, overactive Th17 cell-related cytokines may participate in valve inflammation responses, leading to immune cell infiltration and damage to valve tissues [16]. Previous studies have suggested a link between these cytokines and RHD, with elevated levels of IL-17, IL-6, IL-21, and IL-23 observed in affected patients [17]. This study aims to demonstrate the expressions of Th17 cell-related cytokines in RHD patients and analyze their correlation with heart valve damage. By gaining deeper insights into this relationship, the research provides new perspectives on the pathogenic mechanisms of RHD, paving the way for targeted strategies for early diagnosis and treatment of the disease. Ultimately, this approach is expected to improve the prognosis of RHD patients, alleviate the health burden, and enhance quality of life.

## Materials and methods

Research object

In this study, a total of 20 patients diagnosed with rheumatic heart disease (RHD) who underwent mitral valve replacement surgery were designated as the "RHD Group." The inclusion criteria for this group were carefully outlined to ensure the selection of appropriate candidates. Patients were required to exhibit severe abnormalities in mitral valve structure, as confirmed by echocardiography, alongside experiencing severe symptoms related to their mitral valve disease, such as dyspnea, cardiac murmurs, and angina. Furthermore, participants needed to present with severe rheumatic mitral valve stenosis or insufficiency, making them suitable for surgical intervention while posing manageable surgical risks. Importantly, all individuals were informed about the study and provided written consent for sample collection.

Conversely, the exclusion criteria were equally stringent to refine the focus of the study on RHD patients. Those with non-rheumatic valvular diseases, such as congenital malformations or infective endocarditis, were excluded, as were individuals with coexisting severe cardiac conditions, including coronary artery disease and aortic diseases. Additionally, patients with active infectious diseases, such as pulmonary tuberculosis or sepsis, were not considered for this study. These criteria helped ensure that the study concentrated on the specific impacts of RHD on mitral valve replacement outcomes.

For comparative analysis, a control group, labeled the "Degenerative Valve Disease Group," comprised another 20 patients who underwent mitral valve replacement surgery for non-rheumatic conditions, specifically degenerative mitral valve prolapse. All patients in both groups were validated through echocardiographic assessment. To maintain the integrity of the study, demographic factors, such as age, gender, and comorbidities, were closely matched between the two groups. This careful selection and matching process aimed to bolster the robustness of the findings and facilitate a thorough analysis of Th17 cytokines, exploring their relationship with the structural and functional aspects of valve damage in the context of RHD versus degenerative valve disease.

Cardiac valve sampling

During the surgical procedure, extracorporeal circulation was established for patients, and discarded cardiac valve materials from the surgery were immediately placed in 4% paraformaldehyde for fixation for 16 hours. The rationale for using these discarded valve materials is that they provide valuable insights into the pathological changes associated with RHD. These samples are representative of the overall pathology, reflecting the histological characteristics and inflammatory processes observed in RHD-affected valves, which allows for a more comprehensive investigation of Th17 cell-related cytokine expression. After fixation, the samples were transferred to a decalcification solution to soften the tissues. Decalcification is necessary for valve samples, particularly those with calcification due to chronic disease, as it facilitates the creation of thin sections for histological evaluation. This step is critical to preserving the cellular architecture and obtaining high-quality samples for analysis. We optimized our decalcification protocols to minimize these effects and ensure the reliability of our immunohistochemical results. Following decalcification, routine paraffin embedding was performed, and continuous sections of 4 μm thickness were obtained for both immunohistochemistry staining and HE staining.

Immunohistochemical staining and HE staining

The paraffin-embedded sections were initially subjected to deparaffinization and hydration procedures to render the tissue sections suitable for staining. Antigen retrieval was performed using heat-induced epitope retrieval (HIER) in a sodium citrate buffer (pH 6.0) at 95°C for 30 minutes to restore the immunoreactivity of antigens within the tissue. Following this, 3% H2O2 was introduced to eliminate endogenous peroxidase enzymes in the tissue, thereby reducing nonspecific staining. A working solution of normal goat serum was applied to the sections at 25°C for 15 minutes to minimize nonspecific antibody binding. After serum application, rabbit anti-human interleukin antibodies (IL-17, IL-6, IL-23, IL-21) were used; these antibodies were sourced from Thermo Fisher Scientific and included polyclonal/monoclonal antibodies as appropriate. The sections were incubated overnight at 4°C to allow for effective binding of antibodies to the target antigens of interest. After each antibody incubation step, the slides were thoroughly washed three times with phosphate-buffered saline (PBS) for five minutes each to remove unbound antibodies and other nonspecific binding substances. Diaminobenzidine (DAB) chromogenic substrate was utilized for color development at room temperature, typically requiring seven minutes for optimal staining. This process induced a staining reaction that rendered the target antigens visible. To ensure optimal staining, preliminary experiments were conducted to determine the optimal duration of DAB staining, which confirmed that seven minutes provided the best signal-to-noise ratio. Counterstaining with hematoxylin was carried out to stain cell nuclei, enhancing the visibility of tissue structures. Dehydration and clearing processes were performed to prepare the tissue sections for mounting. The sections were mounted on glass slides for observation and the enumeration of inflammatory cells under a microscope.

Evaluation indicators

Cytokine detection (IL-17, IL-6, IL-23, IL-21) involved visualizing cytoplasmic staining patterns to identify positive reactions, with PBS used as a negative control in place of the primary antibody. Five randomly chosen high-power fields (×400 magnification) were examined, and both total and positive cell counts per field were recorded. Cells were scored based on staining intensity as follows: deep brown (3 points), brown-yellow (2 points), light yellow (1 point), or no staining (0 points). Additionally, scores were assigned based on the proportion of positive cells: 0 points (negativity), 1 point (< 10% positive cells), 2 points (11%-50% positive cells), 3 points (51% to ~75% positive cells), and 4 points (> 75% positive cells). An immunoreactive positive response was confirmed when the percentage of positive cells multiplied by the staining intensity score exceeded three points. The chosen cutoff score of greater than 3 points for defining a positive response was established based on previous literature, indicating that this threshold correlates with significant biological activity and immune response in similar studies. This cutoff was further validated by assessing the positive predictive value against known positive controls within the study cohort. To ensure reliability and minimize bias, scoring was conducted by two independent observers. Inter-observer variability was assessed using Cohen’s kappa coefficient, which demonstrated a high level of agreement between observers (kappa = 0.75). This rigorous scoring process supports the robustness and validity of our findings.

Statistical methodologies

Statistical analyses were performed using Statistical Product and Service Solutions (SPSS, version 23.0; IBM SPSS Statistics for Windows, Armonk, NY). Continuous variables were reported as mean ± standard deviation. Prior to conducting the t-test for mean comparisons, the normality of continuous variables was tested using the Shapiro-Wilk test. If the data were found to be normally distributed, the t-test was utilized for comparisons; otherwise, non-parametric tests, such as the Mann-Whitney U test, were employed. Comparisons for categorical variables were carried out using the chi-square test. The Spearman correlation analysis method was used for correlation analysis, with statistical significance set at P < 0.05. A power analysis was conducted prior to the study to determine the adequate sample size needed to detect significant differences between groups. This analysis suggested that the sample sizes chosen would provide sufficient power (typically set at 0.80) to detect meaningful differences, thereby minimizing the potential for type II errors in our findings.

Ethical considerations

This study was approved by the Institutional Review Board (IRB) of Shalamar Hospital, Lahore, Pakistan. Informed consent was obtained from all participants, ensuring they were aware of the study's purpose, procedures, and potential risks associated with mitral valve surgery and sample collection, which may include bleeding, infection, and anesthesia-related complications, although these risks are minimal. Patient confidentiality was strictly maintained by anonymizing personal information, and data were securely stored in a password-protected database accessible only to authorized personnel. The research adhered to the principles of the Declaration of Helsinki, prioritizing patient welfare and rights. All potential conflicts of interest were disclosed, ensuring integrity and transparency throughout the research.

## Results

IL-17 in mitral valve tissue

**Figure 1:**
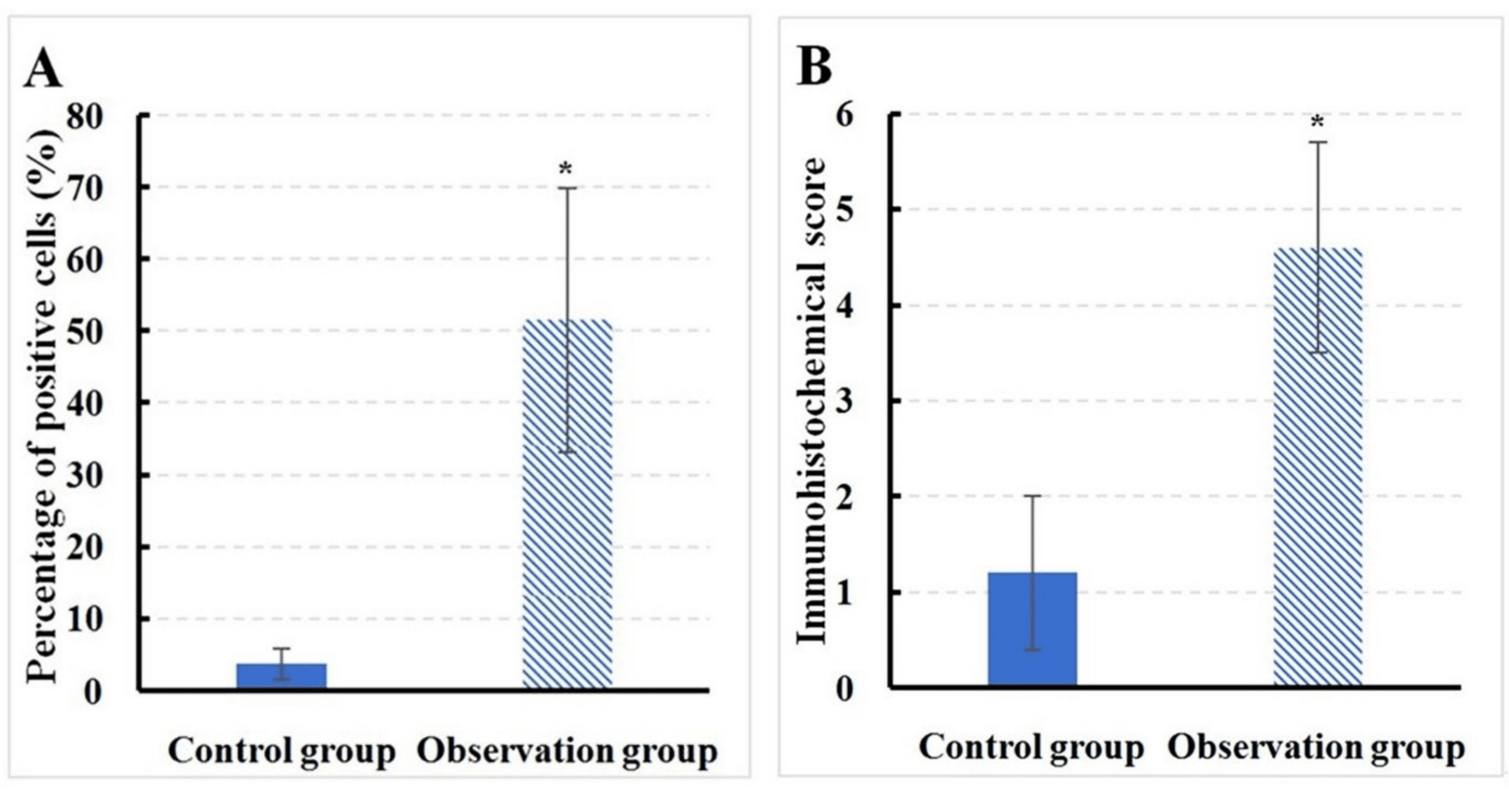
Contrast of IL-17 between groups A: positive cell count; B: immunohistochemical score; *P < 0.05 vs. Group C; χ² = 12.60; p-value = 0.0004 Mean comparisons were conducted using the t-test; comparisons for categorical variables were carried out using the chi-square test.

In Group O, the proportion of IL-17-positive cells in mitral valve tissue was 51.6 ± 18.4%, with an immunohistochemistry score of 3.7 ± 2.2. In contrast, in Group C, the proportion of IL-17 positive cells in mitral valve tissue was 3.7 ± 2.2%, with an immunohistochemistry score of 1.2 ± 0.8. A substantial difference was observed between groups, with IL-17 in the mitral valve tissue of Group O being drastically superior to that in Group C (P < 0.05) (Figure 1). The normality of data distribution was assessed using the Shapiro-Wilk test, and the homogeneity of variances was evaluated with Levene's test. Mean comparisons were conducted using the t-test, with an effect size (Cohen’s d) of 2.28. IL-17 levels in Group O were approximately 51.6%, compared to 3.7% in Group C, indicating a 1,300% increase.

IL-21 in mitral valve tissue

**Figure 2:**
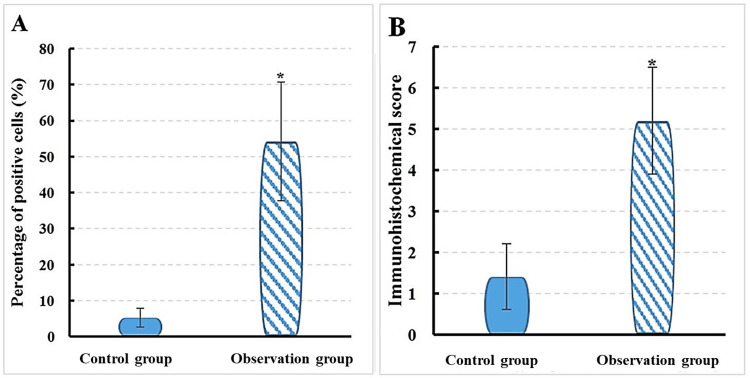
Contrast of IL-17 between groups A: positive cell count B: immunohistochemical score; *P < 0.05 vs. Group C; χ² = 13.79; p-value = 0.0002 Mean comparisons were conducted using the t-test; comparisons for categorical variables were carried out using the chi-square test.

The test results revealed that, in Group O, the proportion of IL-21-positive cells in the mitral valve tissue was 54.2 ± 16.5%, with an immunohistochemistry score of 5.2 ± 1.3. In contrast, in Group C, the proportion of IL-21-positive cells in the mitral valve tissue was 5.3 ± 2.6%, with an immunohistochemistry score of 1.4 ± 0.8. A notable difference was found between groups, with IL-21 in mitral valve tissue of Group O being markedly superior to that in Group C (P < 0.05) (Figure 2). Mean comparisons revealed a substantial difference, with an effect size of 2.87. IL-21 levels in Group O were 54.2% compared to 5.3% in Group C, reflecting a 925% increase.

IL-6 in mitral valve tissue

**Figure 3:**
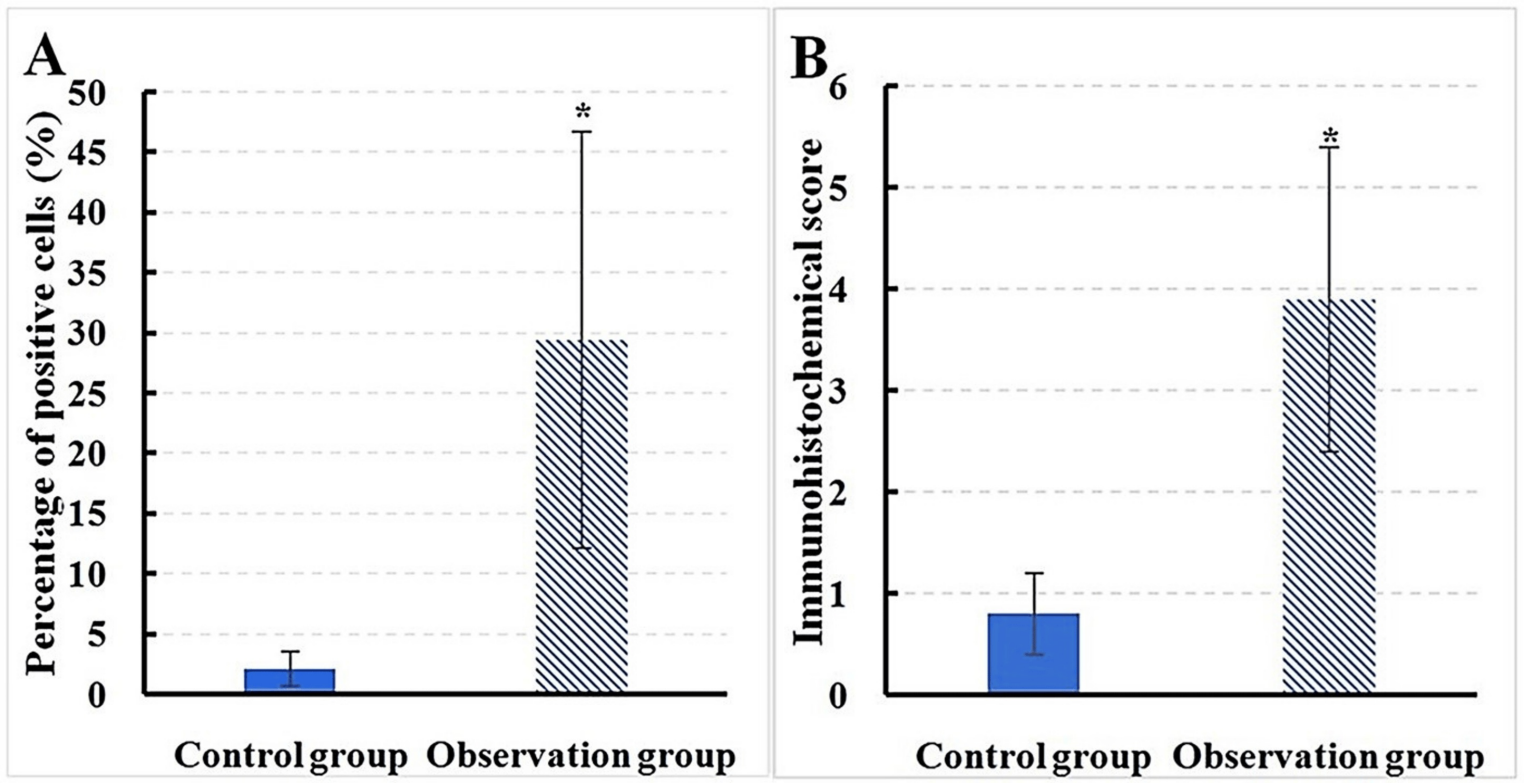
Contrast of IL-6 expression between groups A: positive cell count; B: immunohistochemical score; *P < 0.05 vs. Group C; χ² = 10.82; p-value = 0.001 Mean comparisons were conducted using the t-test; comparisons for categorical variables were carried out using the chi-square test.

The test results revealed that, in Group O, the proportion of IL-6-positive cells in the mitral valve tissue was 29.4 ± 17.3%, with an immunohistochemistry score of 3.9 ± 1.5. In contrast, in Group C, the proportion of IL-6-positive cells in the mitral valve tissue was 2.1 ± 1.4%, with an immunohistochemistry score of 0.8 ± 0.4. A considerable difference was indicated between groups, with IL-6 in mitral valve tissue of the observation group being greatly superior to that in Group C (P < 0.05) (Figure 3). The t-test showed an effect size of 2.40, with IL-6 levels at 29.4% in Group O versus 2.1% in Group C, indicating a 1,300% increase.

IL-23 in mitral valve tissue

**Figure 4:**
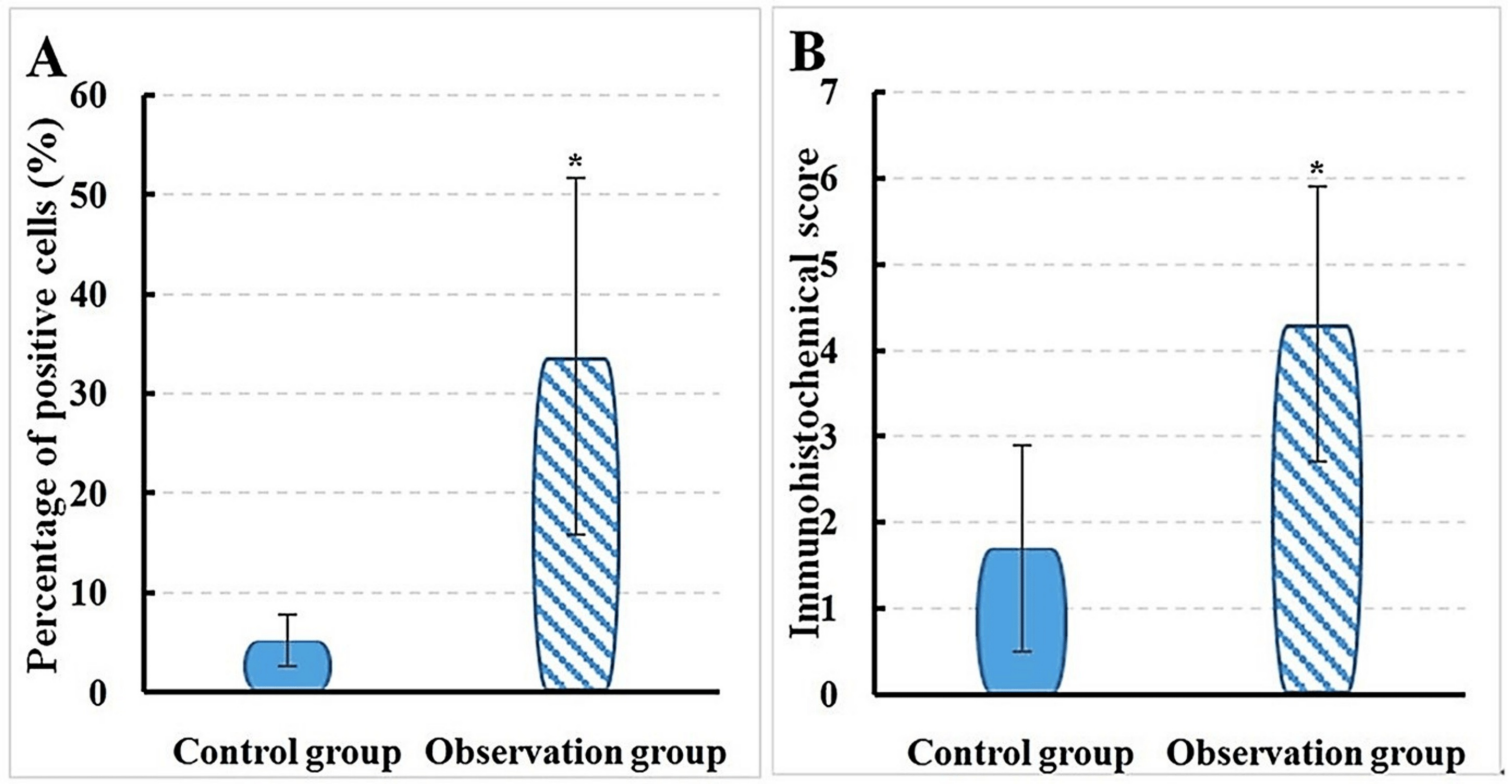
Contrast of IL-23 expression between groups A: positive cell count; B: immunohistochemistry score; *P < 0.05 vs. Group C; χ² = 9.90; p-value = 0.0016 Mean comparisons were conducted using the t-test; comparisons for categorical variables were carried out using the chi-square test

The test results indicated that, in Group O, the proportion of IL-23-positive cells in the mitral valve tissue was 33.7 ± 17.9%, with an immunohistochemistry score of 4.3 ± 1.6. In contrast, in Group C, the proportion of IL-23-positive cells in the mitral valve tissue was 5.2 ± 2.6%, with an immunohistochemistry score of 1.7 ± 1.2. A dramatic difference was found between groups, with IL-23 in mitral valve tissue of Group O being considerably superior to that in Group C (P < 0.05) (Figure 4). An effect size of 2.41 was observed, with IL-23 levels at 33.7% in Group O compared to 5.2% in Group C, indicating a 549% increase.

Analysis of the correlation between IL-17 cytokines and cardiac valve injury

The correlation analysis indicated that the expression of IL-17-related cytokines, including IL-17, IL-21, IL-6, and IL-23, in valve tissue, showed a positive correlation with heart valve damage (P < 0.05) (Table 1). 

**Table 1:** Correlation analysis results Spearman correlation analysis

Cytokine	Correlation coefficient (r)	P
IL-17	0.417	0.003
IL-21	0.625	0.000
IL-6	0.508	0.000
IL-23	0.326	0.008

**Figure 5:**
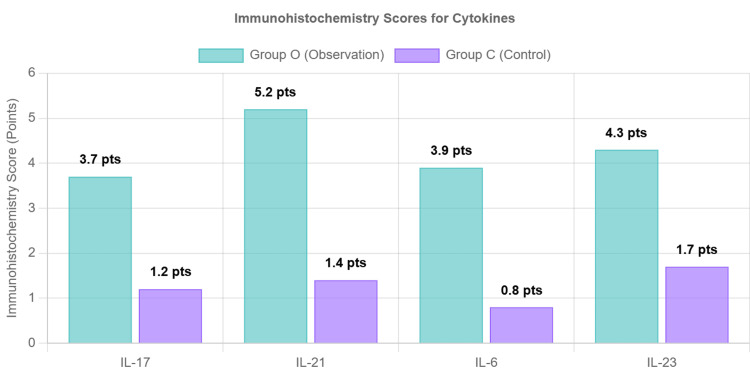
Immunohistochemistry scores for cytokines in mitral valve tissue

Figure 5 illustrates the immunohistochemistry scores for cytokines IL-17, IL-21, IL-6, and IL-23 in the observation group (Group O) and control group (Group C). The scores reflect the intensity of cytokine expression in the tissue samples. For IL-17, the immunohistochemistry score in Group O was around 3.7, significantly higher than the score in Group C, which was approximately 1.2. IL-21 had the highest score in Group O at approximately 5.2, compared to about 1.4 in Group C. The scores for IL-6 and IL-23 in Group O were approximately 3.9 and 4.3, respectively, while, in Group C, they were significantly lower at around 0.8 and 1.7, respectively. This indicates a much stronger expression of these cytokines in Group O compared to Group C.

**Figure 6:**
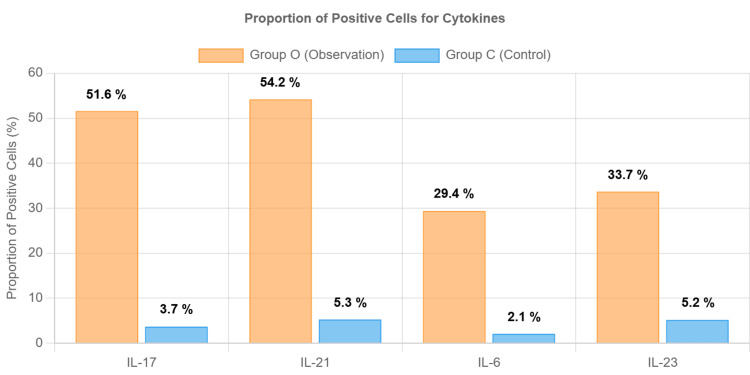
Proportion of positive cells for cytokines in mitral valve tissue

Figure 6 shows the proportion of positive cells for various cytokines IL-17, IL-21, IL-6, and IL-2 in Group O and Group C. In Group O, the proportions of positive cells were markedly higher for all cytokines compared to Group C. Specifically, for IL-17, the proportion of positive cells in Group O was around 51.6%, while it was significantly lower in Group C, at approximately 3.7%. Similarly, for IL-21, Group O exhibited a high proportion of positive cells at about 54.2%, in contrast to the much lower proportion of approximately 5.3% in Group C. IL-6 and IL-23, followed a similar trend, with Group O showing proportions of approximately 29.4% and 33.7%, respectively, whereas Group C showed lower proportions of about 2.1% for IL-6 and 5.2% for IL-23. These differences indicate that the cytokine expression in Group O is significantly elevated compared to Group C.

## Discussion

Heart diseases pose significant challenges in global health, with RHD being a critical subtype that emerges from untreated rheumatic fever. This condition profoundly impacts cardiac structure and function, manifesting through various symptoms, including arthritis, pericarditis, endocarditis, and notably damage to the mitral and aortic valves [16]. Research indicates that valve damage in RHD predominantly results from autoimmune reactions initiated by rheumatic fever. Anti-streptococcal antibodies induce inflammatory responses that lead to immune cell infiltration in the endocardium of heart valves, release inflammatory mediators, and collagen degradation, culminating in valve fibrosis, thickening, and scar formation. These pathological alterations can result in valve stenosis or regurgitation, ultimately causing cardiac dysfunction [17].

In the past, surgical repair or replacement of valves represented the primary treatment option for RHD. However, contemporary treatment strategies encompass a combination of medical therapy, interventional treatments, and surgical procedures. Medical management typically includes prophylactic antibiotics and anti-inflammatory drugs aimed at symptom relief and inflammation control [18]. Interventional approaches, such as balloon dilation and valve replacement, have proven effective in many cases [19]. Despite these advancements, RHD treatment remains challenging, particularly in resource-limited settings, necessitating further research to optimize treatment strategies and improve patient outcomes. Our study explored the correlation between Th17 cell-related cytokines and heart valve damage, providing insights into potential diagnostic and therapeutic avenues for RHD patients.

Our findings demonstrated that the proportion of IL-17-related cytokine-positive cells and immunohistochemistry scores in mitral valve tissues from Group O significantly exceeded those from Group C (P < 0.05). Elevated levels of Th17 cell-related cytokines in RHD patients may contribute to the disease's pathogenesis, characterized by inflammation and cardiac valve damage. Previous research has shown that Th17-related cytokines, such as IL-6 and IL-17, are pivotal in immune signaling pathways and inflammatory responses. For instance, Xian et al. [20] observed elevated IL-6 and IL-17 levels in RHD rat models, correlating with increased valve inflammation and fibrosis. Similarly, Chen et al. [21] reported substantial increases in IL-6 and IL-17 in heart valve tissues and serum, indicating their crucial roles in the autoimmune processes underlying RHD. By inducing inflammation and promoting immune cell infiltration, Th17-related cytokines facilitate structural and functional damage to cardiac valves, underscoring their potential as therapeutic targets [22].

The implications of targeting Th17 cytokines in clinical practice are significant. Therapeutic interventions aimed at modulating the expression of these cytokines, such as immunomodulatory therapies or biologics that inhibit their activity, may alleviate inflammation and mitigate valve damage [23,24]. Existing treatments targeting cytokine pathways, such as IL-6 inhibitors, are already in use for other autoimmune diseases, suggesting potential applicability in RHD management. Moreover, ongoing clinical trials exploring the efficacy of Th17-targeting agents could further validate this approach. However, limitations such as sample size and patient homogeneity in our study restrict the generalizability of our findings. A more diverse cohort could provide insights into varying patient responses to treatments. Future studies should focus on elucidating the underlying molecular mechanisms, such as specific signaling pathways associated with Th17 cells, which might serve as targets for therapeutic intervention. For instance, investigating pathways such as the JAK/STAT signaling cascade could reveal novel strategies for RHD management.

Strengths and limitations

This study presents several strengths and limitations that significantly impact the interpretation of its findings. A notable strength is the rigorous selection criteria for both the RHD Group and the degenerative valve disease group, which ensured that participants had clearly defined diagnoses and comparable demographics. This matching enhances the validity of the findings related to Th17 cytokines and their relationship with valve damage. Additionally, the use of discarded cardiac valve materials allows for a direct examination of the pathological changes associated with RHD, facilitating a comprehensive analysis of cytokine expression through well-established immunohistochemical methods. These findings suggest that targeting Th17 cytokines could influence treatment protocols for RHD, potentially leading to novel therapies aimed at reducing valve damage and improving patient outcomes. These results carry broader implications for public health, particularly in regions where RHD is endemic, highlighting the need for strategies that address cytokine-mediated inflammation.

However, the study is not without limitations. The relatively small sample size of 20 patients in each group may limit the generalizability of the findings, as it restricts the diversity in patient responses to treatment and cytokine expression, which could vary significantly across different demographic backgrounds or disease severities. Certain genetic or environmental factors may influence how patients respond to Th17 cytokine modulation. Moreover, the cross-sectional nature of the study restricts the ability to infer causal relationships between Th17 cytokine expression and the observed valve damage. To further investigate the underlying molecular mechanisms, future research could benefit from employing methodologies such as *in vitro* studies to elucidate cytokine pathways, as well as larger cohort analyses to capture a wider range of patient characteristics and responses. By advancing our understanding of the roles these cytokines play in RHD, this research lays the groundwork for innovative therapeutic approaches and underscores the necessity of continued exploration in both clinical practice and public health initiatives.

## Conclusions

The results of this study demonstrated that Th17 cell-related cytokine expressions (IL-17, IL-21, IL-6, IL-23) were significantly elevated in the heart valve tissues of patients with RHD valve damage, indicating a strong positive correlation with the severity of heart valve damage. The proportion of IL-17-, IL-21-, IL-6-, and IL-23-positive cells in the RHD group was found to be dramatically higher compared to the degenerative valve disease control group, highlighting the critical role of these cytokines in RHD pathology. The finding highlights the potential of these cytokines as therapeutic targets for the treatment and prevention of RHD. Conducting in-depth investigations into the underlying molecular mechanisms, such as in *vitro studies* and larger cohort analyses, will facilitate the identification of more specific therapeutic targets and strategies. Ultimately, a deeper understanding of the roles of these cytokines could pave the way for novel therapies, significantly impacting clinical practice and improving patient outcomes in RHD management.
